# Soil Microbes Drive the Flourishing Growth of Plants From *Leucocalocybe mongolica* Fairy Ring

**DOI:** 10.3389/fmicb.2022.893370

**Published:** 2022-05-20

**Authors:** Qiqi Wang, Chong Wang, Yumei Wei, Weiqin Yao, Yonghui Lei, Yanfei Sun

**Affiliations:** ^1^College of Life Sciences/Xinjiang Production and Construction Corps Key Laboratory of Oasis Town and Mountain-Basin System Ecology, Shihezi University, Shihezi, China; ^2^Ürümqi Customs Technique Center, Ürümqi, China; ^3^Department of Plant protection, College of Agriculture, Shihezi University, Shihezi, China

**Keywords:** fairy ring, high throughput sequencing, plantgrowth-promoting rhizobacteria, salt tolerance, diversity

## Abstract

Fairy ring is a natural phenomenon in which fungal fruiting bodies occur as a ring on a spot. This ring is produced due to spore ejection by Basidiomycetous fungi and forms a lush growing plant belt. However, the drivers for such formations and the potential plant growth-promoting rhizobacteria in fairy ring soils remain unknown. Fairy rings formed by *Leucocalocybe mongolica* were selected in this study. Soil characteristics and microbial (bacteria and fungi) community structures between beneath and outside the fairy rings were compared through high-throughput sequencing. Beneficial bacterial resources were excavated using dependent culturable methods. Soil electrical conductivity and available potassium were higher in the soil beneath the ring than outside it. These parameters were positively correlated with the dominant microbial community, but microbial diversity was lower. In the soil beneath the fairy ring, Bacteroidetes and Basidiomycota were more abundant, whereas Verrucomicrobia was less prevalent. *Bacillus pumilus* (strain BG-5) was isolated from the soil beneath the ring. Strain BG-5 can solubilize phosphorus and produce indole-3-acetic acid, NH_4_^+^, and siderophores. Furthermore, strain BG-5 enhanced salt tolerance and promoted the growth of *Arabidopsis thaliana*, wheat (*Triticum aestivum*), and cotton (*Gossypium hirsutum*) seedlings. This study indicated the presence of abundant beneficial microbes driving the flourishing growth of plants in the fairy ring soil and provided bio-resources for agricultural growth-promoting agents.

## Introduction

Fairy rings are a concentric growth of saprophytic soil Basidiomycetous fungi. These rings are concentric regular dark green grass bands characterized by a strip of luxuriant growth, contradictory to the general or dead vegetation in adjacent zones, formed by fungi. This growth generally moves outward on all directions from the center site and is an evident above-ground regular circle of fruiting bodies (Caesar-Tonthat et al., 2013). More than 60 species of Basidiomycetous fungi can form fairy rings, such as *Lepista sordida, Gymnopus erythropus*, and *Marasmius dryophilus* (Choi et al., 2010; Bonanomi et al., 2013; Falandysz et al., 2014; Karst et al., 2016; Ito et al., 2020). *Leucocalocybe mongolica* a precious wild mushroom rich in protein, vitamins, potassium, calcium, iron, phosphorus, and other minerals and is popular in domestic and foreign markets (Wang et al., 2019; Du et al., 2016). Similar to other basidiomycetes, *L. mongolica* can form the typical fairy ring.

Fairy ring fungi can markedly modify the soil quality, thereby indirectly affecting plant growth. Soil available phosphorus (P) and inorganic nitrogen (N) content is high in the fairy ring formed by fungi, such as *Agaricus arvensis* (Edwards, 1984), *Marasmius oreades* (Fisher, 1977), and *Agaricus campestris* (Bonanomi et al., 2012). These fungi can also enhance organic matter mineralization and accumulate more nutrition in the soil. On the other hand, changes in properties of soil prepared for producing their abundant sporophores provide a beneficial condition for plant growth. Some researchers have found that fairy ring fungi can degrade the soil humus to accumulate NH_4_^+^ and NO_3_^–^ content, and plants growing in such soil showed stimulated growth (Mathur, 1970). In addition to the influence of fairy ring fungi on soil physiochemical properties, some biological factors may contribute to plant growth on this soil. Many reports have demonstrated that *Lepista sordida*, as fairy ring fungi, can produce plant growth-regulating substances, such as 2-azahypoxanthine (AHX), imidazole-4-carboxamide (ICA), and 2-aza-8-oxohypoxanthine (AOH), thereby promoting the growth of rice, wheat, and other crop seedlings and increasing their yield (Kawagishi, 2019; Ito et al., 2020). The vegetation on fairy ring in different zones exhibited different growth states. These fairy rings may also attribute to changes in microbial community niche and the function of some microbial species (Bonanomi et al., 2012; Marí et al., 2020). Soil microorganisms play an essential role in soil fertility, carbon and nitrogen cycling, and organic matter decomposition (Joa et al., 2014). Mycorrhizal fungi, such as arbuscular mycorrhizal and ectomycorrhizal fungi, can form mutually beneficial symbiotic relationships with plants, improve nutrient acquisition with their hyphal network, and enhance plant resistance (Akib et al., 2019). Furthermore, saprotrophic fungi have been reported to play roles in biodegradation or transformation of organic and inorganic pollutants through enzymatic degradation, biochemical or physical immobilization mechanisms, and increasing the availability of soil nutrients (Jennifer et al., 2013; Ito et al., 2020). Plant growth-promoting rhizobacteria (PGPR) interact with the plant rhizosphere, increase the root surface area, and improve nutrient utilization. Some of them can generate siderophores to enhance plant disease resistance or secrete phytohormone (such as indole-3-acetic acid, IAA) to promote plant growth (Pal et al., 2019; Murgese et al., 2020). Ashry et al. (2022) isolated *Bacillus cereus* DS4, that can improve the tolerance of drought sensitive of maize under drought stress and promote plant growth by producing IAA, extracellular polysaccharide, iron carrier and a variety of antioxidant enzymes. Many studies indicated that *Bacillus atrophaeus* can enhance the growth of *Arabidopsis thaliana* and durum wheat under NaCl-stress condition by phosphate solubilization, hydrogen cyanide (HCN), NH_3_ and IAA production, nitrogen fixation, antifungal activity (Kerbab et al., 2021). In addition, some PGPR can indirectly promote plant growth by inhibiting plant pathogens, which are widely prepared into biocontrol agents. *Stenotrophomonas malthopilia* KJKB5.4, *Stenotrophomonas pavanii* LMTSA5.4, *Bacillus cereus* AJ34 and *Alcaligenes faecalis* AJ14 have rhizobacterial antibiosis to against *Curvularia lunata* by producing antibiotic compounds and increasing plant growth (Rahma et al., 2021). The inoculation of *Azospirillum* Er-20 and *Agrobacterium Ca-18* significantly improved the growth and yield of pepper, and had the potential to lower 25% N requirement for chili by nitrogen fixing and phosphorous solubilizing (Raza et al., 2021). Xu et al. (2022) found that the PGPR (*Tsukamurella tyrosinosolvens* P9 and *Burkholderia pyrrocinia* P10) inoculations enhanced the photosynthetic performance, activated the antioxidant defense system, and decreased the ROS level of peanut plants, which alleviated the salt-induced cell damage and enhanced the salt tolerance and growth of peanut. Researchers combined biochar and PGPR (*Azotobacter chroococcum* SARS 10 and *Pseudomonas koreensis* MG209738) to alleviate the adverse impacts of saline water on the growth, physiology, and productivity of maize (*Zea mays* L.), as well as promot the soil properties and nutrient uptake (Nehela et al., 2021). Furthermore, Yu et al. (2022) found that AMF (*Funneliformis mosseae*) and PGPR (*Bacillus megaterium*) had synergetic effects on root morphology, soil nutrient availability, and *Elymus nutans* growth. But whether exist a large number of PGP microbial resources in the fairy ring soil to drive the plant flourishing growth is rarely reported.

However, why plants on fairy rings grow more luxuriantly than the outer area of the ring? What was the force driving the lush plant growth? Until now, the fairy ring-forming fungi–soil properties–soil microorganism–plant interaction and the driving forces that lead to flourishing plant growth on fairy rings remain unclear. Therefore, the motivation of this study was too deeply investigate these factors that driving the lush growth of fairy ring plants. The objectives of this study were (1) to compare the differences of soil physicochemical properties between the different areas of fairy ring caused by *L. mongolica*; (2) to compare the differences of soil microbial community structure and diversity between the different areas of fairy ring caused by *L. mongolica*; (3) to explore the correlation between soil microorganisms and soil properties in fairy ring; (4) to excavate potential PGP microorganisms in fairy ring soil. This study provided a theoretical basis for exploring the driving factors of the lush growth of fairy ring plants, and bio-resources for agricultural growth-promoting agents.

## Materials and Methods

### Study Site Description

The study was conducted in the third pasture of the Corps 12 Division 104th Regiment (85°47′E, 42°55′N) in the northern part of Xinjiang, China. This region has a temperate continental arid climate and an altitude of 3468.93 m. The mean annual temperature and precipitation at this site is −2.56°C and 24.89 mm, respectively. The minimum monthly mean air temperature is −23.81°C in November, and the maximum value is 17.58°C in August (NOAA-Climate Prediction Center^1^). The mean annual relative humidity (2 m from the surface) is 58.04%, and the mean annual surface pressure is 665.23 hPa (Global Modeling and Assimilation Office, 2015), MERRA-2 inst1_2d_asm_Nx: 2d,1-Hourly, Instantaneous, Single-Level, Assimilation, Single-Level Diagnostics V5.12.4, Greenbelt, MD, United States, Goddard Earth Sciences Data and Information Services Center, http://doi:10.5067/3/Z173KIE2TPD). The dominant plant species in the area belonged to *Festuca* (i.e., *F. ovina*) and *Artemisia*. The soil belongs to Tianshan meadow light soil, clay loam. There is a grass felt layer with a thickness of about 10cm on the surface. The content of organic matter is 5%--7%, soil pH is 7.0. the cation exchange capacity is about 40 me/100 g soil (Geographic Data Sharing Infrastructure, College of Urban and Environmental Science, Peking University^2^).

### Soil Sampling Site and Characteristic Analyses

**FIGURE 1:**
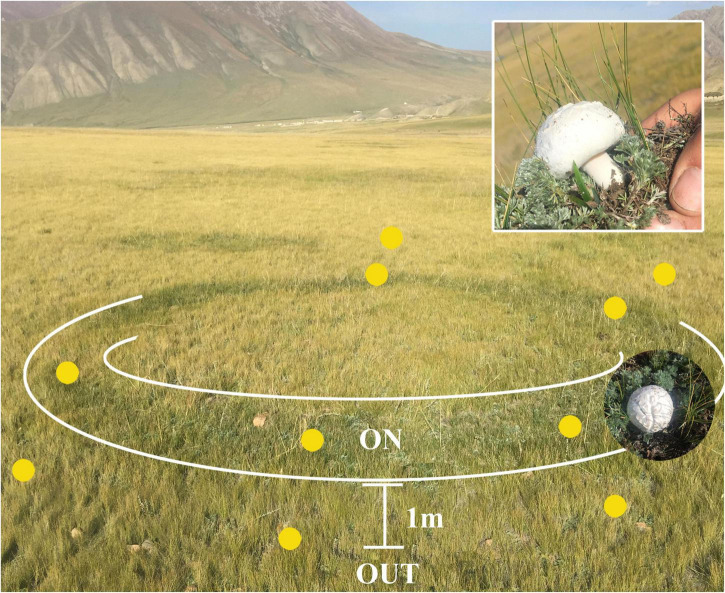
Distribution diagram of soil sampling points from *Leucocalocybe mongolica* fairy ring. ON, the zone located in plant belt that flourishing growth and beneath the fairy ring; OUT, the zone far away from ON zone about 1 m and out of fairy ring, that was not affected by fairy ring fungi. The insert pictures, *Leucocalocybe mongolica* that was fairy ring-forming fungus, and grown in the ON zone.

A total of 12 soil samples were collected from two *L. mongolica* fairy ring (ring 1 and ring 3) zones during October 2017 when the fungus was producing fruiting bodies (Figure 1). The *L. mongolica* fairy rings were similar in size (diameter: approximately 10 m) and were approximately 100 m apart. At each ring, the top soil (0–20 cm) was collected from two zones after removing the thin aboveground litter layer: (1) OUT (including Q1W belonging to ring 1 and Q3W belonging to ring 3), 1 m from the fungal fruiting bodies outside the ring, and this zone was not affected by *L. mongolica* and (2) ON (including Q1 and Q3), beneath the fairy ring, where an abundant mat of white mycelia and fruiting bodies were evident on the top soil. The five-point sampling method was used for each ring, and the collected patterns were mixed and divided into three parts as three replicate soils in every zone. The ON zone soil samples were marked as Q1-1, Q1-2, and Q1-3 (belongs to Q1) and Q3-1, Q3-2, and Q3-3 (belongs to Q3). OUT soil samples were marked as Q1W-1, Q1W-2, and Q1W-3 (belongs to Q1W) and Q3W-1, Q3W-2, and Q3W-3 (belongs to Q3W). Soil samples (5-15 cm depth) were packed in sterile ziplock bags using soil core sampling tool. The samples were quickly transferred in ice bags to the laboratory where they were sieved through a 2mm mesh. Then, the soil samples were separated into three parts: one part of the samples were air dried and stored at room temperature for analyzing soil physicochemical properties; soil water content (SWC), electric conductivity (EC), pH, total nitrogen (TN), total phosphorus (TP), available potassium (AK), and soil organic matter (SOM) were measured using previous methods (Bao, 2000). The second part was stored at −80°C and used for high-throughput sequencing. The third part was stored at 4°C and used for isolating beneficial bacteria.

### DNA Extraction, PCR Amplification, and Gene Clone Library Construction

DNA was extracted from soil samples using the EZNA Stool DNA Kit (Omega Bio-tek, Norcross, GA, United States) according to the manufacturer’s protocols. PCR using target-specific primers 341F (5′-CCTACGGGNGGCWGCAG-3′) and 806R (5′-GGACTACHVGGGTATCTAAT-3′) for the V3–V4 region amplified of bacterial 16S rRNA gene (Niem et al., 2020), and that using the primers ITS3_KYO2F (5′-GATGAAGAACGYAGYRAA-3′) and ITS4R (5′-TCCTCCGCT TATTGATATGC-3′) for the ITS2 region amplified of the fungi eukaryotic ribosomal RNA gene (Lemons et al., 2017), where the barcode is an eight-base sequence unique to each sample. PCR reactions were performed in triplicate using the 50 μL mixture. The amplicons were extracted from 2% agarose gels and purified using the AxyPrep DNA Gel Extraction Kit (Axygen Biosciences, Union City, CA, United States) according to the manufacturer’s instructions and quantified using QuantiFluor-ST (Promega, United States).

### Quality Control, Reads Assembly, and Bioinformatics Analysis

Purified amplicons were pooled in equimolar amounts and paired-end sequenced (2 × 250) on an Illumina platform according to the standard protocols. The raw reads were deposited into the NCBI Sequence Read Archive database. Raw data containing adapters or low-quality reads would affect the following assembly and analysis. Paired-end clean reads were merged as raw tags using FLASH (v 1.2.11) (Tanja and Salzberg, 2011) with a minimum overlap of 10 bp and a mismatch error rate of 2%. Noisy sequences of raw tags were filtered using the QIIME (V1.9.1) (Caporaso et al., 2010) pipeline under specific filtering conditions to obtain high-quality clean tags. The clean tags were searched against the reference database^3^ to perform reference-based chimera checking by using the UCHIME algorithm^4^. All chimeric tags were removed, and finally obtained effective tags were used for further analysis. Chloroplast and mitochondrial sequences were removed from all samples. These effective tags were clustered into operational taxonomic units (OTUs) of ≥97% similarity by using the UPARSE pipeline (Edgar, 2013). The tag sequence with highest abundance was selected as a reprehensive sequence within each cluster. Between-group Venn analysis was performed in R to identify unique and common OTUs. The representative sequences were classified into organisms by a naive Bayesian model by using the RDP classifier (Version 2.2) (Wang, 2007) based on the SILVA database^5^ and UNITE database^6^. The abundance statistics of each taxonomy and phylogenetic tree were constructed in a Perl script and visualized using SVG. Biomarker features in each group were screened using Metastats and LEfSe software.

Alpha diversity indices were calculated in QIIME. The OTU rarefaction curve was also plotted in QIIME. The statistics of between-group alpha index comparison was calculated using Welch’s *t*-test and a Wilcoxon rank test in R. An unweighted UniFrac distance matrix was generated using QIIME. The principal component analysis (PCA) of weighted UniFrac was calculated and plotted in CANOCO 4.5. The Adonis test was performed using R. Correlations between soil microbial compositions and soil characteristics were determined using the redundancy analysis (RDA) tool created by CANOCO (Yu et al., 2020). The correlation between soil physicochemical properties and microorganisms of the fair ring was analyzed using the Monte Carlo test.

### Isolation of Plant Growth-Promoting Rhizobacteria From Fairy Ring Soil

Nutrient broth medium (beef extract 0.3%, peptone 10%, NaCl 0.5%, pH 7.0) was used to isolate and purify bacteria from the fairy ring soil. The obtained strains were tested for their plant growth-promoting (PGP) function (Khan et al., 2020; Mukhtar et al., 2020).

(a) nitrogen fixation, Ashby nitrogen-free medium (KH_2_PO_4_ 0.02%, H_2_SO_4_ 0.02%, NaCl 0.02%, CaCO_3_ 0.5%, Mannitol 1%, CaSO_4_ 0.01%, agar, 1.8%, pH, 7.0) was used to detect nitrogen fixation activity.

(b) phosphate solubilization, Pikovskaya’s Agar (HiMedia) plates was used to detected phosphate solubilization activity of isolated bacterial strains (Pikovskaya, 1948). The inoculum placed in 30°C for 2 days and then observed for the appearance of a clear halo zone around the colonies due to solubilization of inorganic phosphate by organic acid produced by bacteria.

(c) potassium dissolution detection: Aleksandrov’s agar medium was used to detected potassium dissolution activity of isolated bacterial strains, that made the screening medium exhibiting cleared zone of potassium solubilization (Hu et al., 2006).

(d) NH_4_^+^ production detection: fresh peptone water was used to test the production of ammonia by the isolate. 48 h old culture of each isolates was inoculated in 10 ml of peptone water and incubated at 30°C for 72 h. 0.5 ml of Nessler’s reagent was added in each tube and after few minutes yellow to brown precipitate appeared which indicates the production of ammonia (Gusmiaty et al., 2019).

(e) IAA production detection: The selected strains were cultured in the medium supplemented with L-tryptophan and the supernatant was collected. A total of 1.5 ml of the supernatant and 4 ml of Salkowski reagent (12 g of FeCl_3_ per liter in 7.9 mol⋅L^–1^ H_2_SO_4_) were mixed. Then, the solution mixture was incubated in the dark for 24 h. The intensity of the resulting pink color was measured at a wavelength of 520 nm using a spectrophotometer. The IAA concentration was determined using a standard curve of known concentrations of IAA (Gusmiaty et al., 2019).

(f) siderophore production: the chrome azurole S agar (CAS) plates were prepared and spot inoculated with test organism and incubated at 37°C for 48 h. Development of yellow-orange halo around the colony was considered as positive for siderophore production (Clark and Bavoil, 1994).

### Evaluation of the Plant Growth-Promoting Effects of Selected Bacterial Strain on Different Plants

#### Plant Material, Growth Conditions, and Salt Treatment

*Arabidopsis thaliana*, wheat, and cotton seeds were used to explore the responses of these plants to salt stress after selected bacterial strain inoculation. The seeds were washed with sterile water for 3 times and soaked in 70% ethanol (v/v) 30s, sterile water washed seeds 5 times again, and subsequently soaked in 2% sodium hypochlorite 5–8 min, finally, sterile water washed seeds 5 times to complete the surface sterilized. After sterilization, the seeds were kept in sterile, moist petri dishes for germination to ensure synchronized growth. *A. thaliana* seeds in the same growth state were transferred to 1/2 MS medium solid plates with 0, 50, and 100 mmol⋅L^–1^ NaCl, and 8 seeds were planted 2 cm from the center of the plates (Table 1). The *A. thaliana* plate was cultured at 24°C for 16 h during the day and at 18°C for 8 h during night. All of six treatments were performed, see Table 1. The CK group included plants with no selected bacterial strain inoculation. The growth status of *A. thaliana* was observed after 30 days.

**TABLE 1:** The treatments of three plants were no-/inoculated with selected bacterial strain under different concentrations of salt stress.

Plant	Salt treatment (NaCl)	PGPR treatment (selected bacterial strain)
*Arabidopsis thaliana*	0 mmol⋅L^–1^	Non-inoculated
	50 mmol⋅L^–1^	Non-inoculated
	100 mmol⋅L^–1^	Non-inoculated
	0 mmol⋅L^–1^	Inoculated
	50 mmol⋅L^–1^	Inoculated
	100 mmol⋅L^–1^	Inoculated
Wheat	0 mmol⋅L^–1^	Non-inoculated
(*Triticum aestivum*)	200 mmol⋅L^–1^	Non-inoculated
	0 mmol⋅L^–1^	Inoculated
	200 mmol⋅L^–1^	Inoculated
Cotton	0 mmol⋅L^–1^	Non-inoculated
(*Gossypium hirsutum*)	150 mmol⋅L^–1^	Non-inoculated
	300 mmol⋅L^–1^	Non-inoculated
	0 mmol⋅L^–1^	Inoculated
	150 mmol⋅L^–1^	Inoculated
	300 mmol⋅L^–1^	Inoculated

To prepare the bacterial suspension, the selected bacterial strain was inoculated into nutrient broth medium and cultivated at 30°C and 180 rpm for 24 h for activation. The seeding bacterial cultures were inoculated into the nutrient broth medium at a ratio of 1% and cultured for 24 h at 30°C and 180 rpm to attain an OD_600_ = 1.0, and that was prepared for inoculation into plant.

The wheat and cotton seeds with uniform growth were placed in pots (3 seeds/pot) filled with nutrient soil and vermiculite (3:1 v/v) under the natural condition. Then, 50 mL of bacterial solution (for bacterial treatment) or sterile water (control treatment) was added every 7 days. Salt stress was induced in wheat and cotton with 0 and 200, and 0, 150, and 300 mmol⋅L^–1^ NaCl, respectively. All plants received equal volume of NaCl solution, without leaching. Overall, four treatments were applied to wheat and six treatments were applied to cotton (Table 1), each treatment had six pots. At 30 and 45 days after wheat and cotton growth, respectively, plant biomass and physiological parameters were evaluated.

#### Plant Growth and Physiological Measurements

For wheat, shoot and root length, fresh and dry weight, Chlorophyll and proline content were detected. For cotton, leaves area, shoot and root length, Chlorophyll, proline and peroxidase content were detected. At the end of the experiment, the soil surface to the apex of the seedling using a scale as shoot length and the underground part were measured as root length. The impurities on the root surface was washed with tap water, the excess water was and absorbed with absorbent manuscript. The whole plants were weighed as fresh weight, and then put them in the 60°C oven to constant weight as dry weight fresh weight. Each treatment was performed in three replicates.

Leaves area testing: The outline of leaves were drawn on the small square manuscript of the standard calculation (the size of the small square was 1 mm × 1 mm), the number of small squares occupied by leaf contour was counted (if it reaches or exceeds half a lattice, it will be counted as one lattice, and if it is less than half a lattice, it will be rejected), the leaves area specified by the grid was obtained.

Chlorophyll content detection: fresh leaves (0.5 g) were thoroughly homogenized in 80% acetone (10 mL) and centrifuged (10,000 rpm) for 15 min. Supernatants were isolated in clean test tubes with 80% acetone (4.5 mL). Samples absorbance was taken at 645 and 663 nm using spectrophotometer (Shimadzu, Japan) (Hussain et al., 2021) Each treatment was performed in three replicates.

Proline content detection: 0.1 g of fresh leaf tissue was put into 1 mL sulfosalicylic acid (SA), mixtures were heated at 95°C for 10 min, then added with acidic ninhydrin in water bath at 95°C for 30 min, after cooling, toluene was added for extraction. The absorbance of reaction solution was read at 520 nm using the spectrophotometer (He et al., 2021). Each treatment was performed in three replicates.

peroxidase (POD) content detection: leaf samples (1 g) were homogenized in 2 mL of 0.1 mol⋅L^–1^ phosphate buffer, pH 7.0 at 4°C. The homogenate was centrifuged at 16 000 g at 4°C for 15 min and the supernatant was used as enzyme source. The reaction mixture consisted of 1.5 mL of 0.05 m pyrogallol, 0.5 mL of enzyme extract and 0.5 mL of 1% H_2_O_2_. The reaction mixture was incubated at room temperature (28 ± 2°C). The changes in absorbance at 420 nm were recorded at 30 s intervals for 3 min. The enzyme activity was expressed as changes in the absorbance min^–1^ mg^–1^ protein (Gemes et al., 2017; Yasmeen et al., 2020). Each treatment was performed in three replicates.

#### Identification of Selected Plant Growth-Promoting Bacterial Strain

The genomic DNA of the selected bacterial strain was extracted using the CTAB method (Alshehri, 2020), and bacterial universal primers 27F (5′-AGAGTTTGATCCTGGCTCAG-3′) and 1492R (5′-GGTTACCTTGTTACGACTT-3′) were selected for PCR amplification of its 16S rRNA gene (Xa and Nghia, 2020). DNAMAN software was used for splicing and assembly of the gene sequences obtained. www.ezbiocloud.net was used to sequence blast with type strains. MEGA 7.0 was used to construct the phylogenetic tree using the neighbor joining method. The sequence information was uploaded to NCBI to obtain the accession number.

### Statistical Analysis

All data were analyzed using SPSS 25.0 (IBM, New York, United States) The independent sample *t*-test and one-way ANOVA, followed by the Duncan test were performed to determine significant differences in related parameters among different samples.

## Results

### Analysis of Soil Physiochemical Properties

*Leucocalocybe mongolica* significantly affected soil physiochemical properties, and soil physiochemistry varied between the ON and OUT zones. EC (*p* = 0.000) and AK (*p* = 0.013) content was significantly higher in the ON zone (60.5% and 15.4%, respectively) than in the OUT zone. By contrast, pH (*p* = 0.011) and SWC (*p* = 0.013) of the soil samples were significantly reduced in the ON zone compared with the OUT zone. No significant difference in TN, TP, and SOM was noted between the ON and OUT zones (Table 2).

**TABLE 2:** Soil physical and chemical properties of soil samples from the fairy ring of *Leucocalocybe mongolica.*

Samples		EC (mS cm^–1^)	pH	SWC (%)	Total P (g kg^–1^)	SOM (g kg^–1^)	Total N (g kg^–1^)	Available K (mg kg^–1^)
ON Zone	Q1	0.754 ± 0.17 a	6.342 ± 0.95a	0.114 ± 0.01 ab	1.580 ± 0.07 ab	177.779 ± 54.97 a	5.264 ± 0.94 a	870.237 ± 99.04 ab
	Q3	0.671 ± 0.02 a	6.209 ± 0.09a	0.112 ± 0.01 b	1.344 ± 0.16 c	173.923 ± 4.83 a	5.137 ± 0.32 a	898.316 ± 137.56 a
	**Average**	**0.713 ± 0.12 A**	**6.276 ± 0.61 B**	**0.113 ± 0.00 B**	**1.462 ± 0.17 A**	**175.851 ± 34.96 A**	**5.201 ± 0.63 A**	**884.277 ± 108.30 A**
OUT zone	Q1W	0.314 ± 0.04 ab	7.197 ± 0.18a	0.117 ± 0.00 ab	1.724 ± 0.14 a	205.059 ± 40.19 a	5.741 ± 0.57 a	696.170 ± 81.72 b
	Q3W	0.252 ± 0.01 b	6.969 ± 0.06a	0.122 ± 0.00 a	1.393 ± 0.05 bc	218.40 ± 13.45 a	5.592 ± 0.19 a	760.386 ± 31.61 ab
	**Average**	**0.283 ± 0.04 B**	**7.083 ± 0.17 A**	**0.119 ± 0.00 A**	**1.559 ± 0.21 A**	**211.730 ± 27.78 A**	**5.667 ± 0.39 A**	**728.278 ± 65.64 B**

*OUT, outside the fairy ring; ON, on the fairy ring. the lowercase letters indicate the difference of the same environmental factor among Q1, Q3, Q1W and Q3W, based on one-way ANOVA followed by Duncan test. Uppercase letters indicate the difference of the same environmental factor between ON zone and OUT zone, based on independent samples t-test. Statistical significant difference was indicated by different letters when P < 0.05. SWQC, soil water content; SOM, Soil organic matter.*

### General Soil Microbial Composition

According to the final sequencing analysis, 1161114 and 1087143 high-quality reads were obtained from bacteria and fungi, respectively. After quality control, filtration, and removal of chimeras, a series of effective tags were obtained. The number of total bacterial tags ranged from 84203 to 93374 and total fungal tags ranged from 67290 to 109569 (Table 3). The OTU rarefaction curves showed that the sequencing depth had basically covered all species in the sample (Supplementary Figure 1). These effective sequences were clustered to 11775 OTUs of bacteria and 1105 OTUs of fungi at the 97% similarity level. The results clearly demonstrated a larger number of OTUs, irrespective of bacteria or fungi, in the OUT samples than in the ON samples (Table 3).

**TABLE 3:** The statistics analyze of sequences information and alpha diversity index of bacteria and fungi from ON and OUT zone of *Leucocalocybe mongolica* fairy rings.

	Samples	Total Tags	Unique Tags	Numbers of OTU	Chao1	Ace	Shannon	Simpson
Bacteria	Q1	91152 ± 1163a	76876 ± 3377a	4421 ± 567a	4421.00 ± 567.00a	5623.85 ± 607.28ab	9.74 ± 0.31b	0.99 ± 0.00b
	Q3	84203 ± 13250a	71163 ± 11458a	4332 ± 406a	5635.46 ± 491.99a	5513.09 ± 420.61b	9.78 ± 0.33b	0.99 ± 0.00b
	Q1W	93374 ± 93374a	81421 ± 12300a	5009 ± 656a	6656.98 ± 4.24a	6529.61 ± 80.57a	10.45 ± 0.03a	0.99 ± 0.00a
	Q3W	88078 ± 15161a	78606 ± 13706a	5134 ± 562a	6475.19 ± 661.47a	6324.39 ± 632.76ab	10.30 ± 0.08a	0.99 ± 0.00ab
Fungi	Q1	67290 ± 4546b	9981 ± 1221c	337 ± 56b	408.79 ± 51.99b	417.75 ± 55.31b	1.66 ± 0.71b	0.38 ± 0.24b
	Q3	74762 ± 7870ab	13705 ± 2161bc	478 ± 113ab	562.79 ± 123.39ab	569.28 ± 115.90ab	2.58 ± 0.68b	0.54 ± 0.15b
	Q1W	109050 ± 23673a	24819 ± 5324ab	548 ± 70a	666.34 ± 96.36a	678.00 ± 89.83a	5.54 ± 0.57a	0.93 ± 0.04a
	Q3W	109569 ± 33027a	26730 ± 10659a	588 ± 155a	648.78 ± 186.04ab	652.68 ± 188.00ab	5.62 ± 0.25a	0.94 ± 0.01a

*The different letters indicate the statistical significant difference (P < 0.05) among different samples of same items and species, based on one-way ANOVA followed by Duncan test.*

Venn diagrams showed that the number of bacteria-unique OTUs obtained from Q1, Q3, Q1W, and Q3W were 980, 754, 1383, and 1009, respectively, and 2364 common OTUs were detected in all samples (Supplementary Figure 2A). The number of fungi-unique OTUs from Q1, Q3, Q1W, and Q3W were 53, 62, 134, and 169, respectively, and 273 common OTUs were detected in all samples (Supplementary Figure 2B).

The alpha diversity index reflected the diversity and richness in different samples. For bacteria, the Chao 1 and Ace index was slightly higher in Q1W and Q3W than in Q1 and Q3. Consistent with Chao 1, the Shannon index was also higher in the OUT zone (Table 3). For fungi, Q1W and Q3W had a significantly higher Shannon index and a significantly lower Simpson index than Q1 and Q3 (*p* < 0.05). In addition, Q1W and Q3W had higher Chao 1 and ACE indices than Q1 and Q3 (Table 3). This indicating that the bacterial and fungal richness and diversity were higher in the OUT zone (Q1W and Q3W) than in the ON zone (Q1 and Q3).

### Effect of Fairy Ring Fungi on Bacterial and Fungal Community Composition and Diversity

**FIGURE 2:**
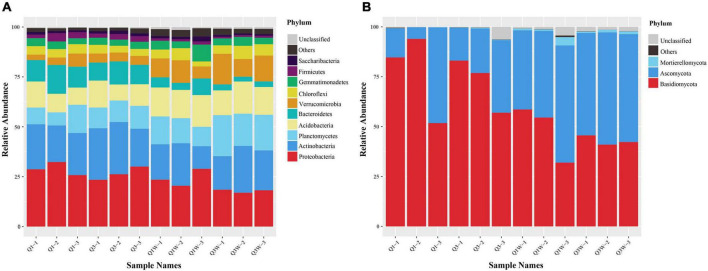
Relative abundance of bacteria and fungi in phylum level. Weighted UniFrac UPGMA tree based on V3-V4 16S rRNA gene of bacteria and ITS2 DNA sequences of fungi obtained from 12 different samples related with fairy rings OUT zone and ON zone. **(A)** bacteria; **(B)** fungi.

The 11775 bacterial OTUs were assigned to 41 phyla, 105 classes, 143 orders, 273 families, and 423 genera, and the 1105 fungal OTUs were assigned to 8 phyla, 21 classes, 58 orders, 99 families, and 140 genera. Proteobacteria and Actinobacteria were the dominant phyla in the ON and OUT zones and constituted an average of 27.78% and 21.09% and 22.14% and 18.45% of the total bacterial population in the ON and OUT zones, respectively; their richness was relatively stable. Abundance of Firmicutes and Bacteroidetes were higher, and Verrucomicrobia decreased extremely in the ON zone than in the OUT zone. Bacteroidetes were more active in the ON zone. In addition, Planctomycetes and Acidobacteria occupied a crucial position (Figure 2A).

The 1105 fungal OTUs were assigned to 8 phyla, 21 classes, 58 orders, 99 families, and 140 genera. Compared with the bacterial community, the fungal community was less diverse. The fungal phyla Basidiomycota (85.04%) and Ascomycota (25.22%) seemed to act as major players in all samples evident through their high relative abundance and frequency (Figure 2B). Among all the samples, 9212 sequences were clustered as 20 OTUs, classified into Glomeromycota, categorized into 6 genera, and belonged to arbuscular mycorrhizal fungi (AFM). The results showed a higher AMF richness and diversity in the OUT zone than in the ON zone. However, due to the low abundance of sequences (abundance <0.1%), they were categorized into “Others” (Figure 2B). The abundance of Ascomycota was lower in the ON zone (Q1 and Q3) than in the OUT zone (Q1W and Q3W), but that of Mortierellomycota reached an obviously low level, such that it nearly disappeared (Figure 2B).

**FIGURE 3:**
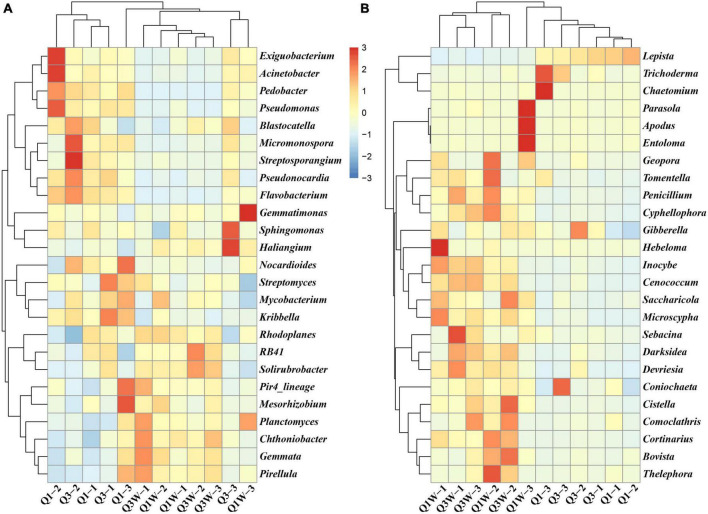
Heatmap displaying the relative abundances of the most dominant genus, the relative abundance of species in at least one sample is above 0.1% (top 25) in each sample. The dendrogram represents complete-linkage agglomerative clustering, based on Euclidean dissimilarities. **(A)** bacteria; **(B)** fungi.

The heatmap analysis presented the top 25 domain bacteria and fungi at the genus level of the 12 samples from the OUT and ON zones (Figure 3). For bacteria, the domain cluster genera were *RB41* (11.19%), *Pedobacter* (4.57%), *Sphingomonas* (4.47%), *Pseudonocardia* (3.68%), *Nocardioides* (3.45%), *Gemmata* (3.45%), *Gemmatimonas* (3.06%), *Chthoniobacter* (2.97%), *Pseudomonas* (2.81%), and *Exiguobacterium* (2.53%) (Figure 3A). These genera seemed to act as major players in all samples evident through their high abundance. However, significant difference was observed among Q1, Q3, Q1W, and Q3W. The abundance of *Pedobacter, Pseudonocardia, and Pseudomonas* was higher in Q1 and Q3 than in Q1W and Q3W, but that of *Gemmata* and *RB41* was lower in Q1 and Q3 than in Q1W and Q3W. For fungi, *Lepista* (42.06%), *Inocybe* (18.10%), *Trichoderma* (8.15%), and *Cenococcum* (7.50%) were the domain genera in all samples. Considerable differences were observed between Q1 and Q3 and Q1W and Q3W (Figure 3B). The abundance of *Inocybe* and *Cenococcum* was significantly lower in Q1 and Q3 (2.87% and 0.92%, respectively) than in Q1W and Q3W (18.1% and 7.4%, respectively). Conversely, the abundance of *Trichoderma* was significantly higher in Q1 and Q3 (13.54%) than in Q1W and Q3W (3.14%). The diversity and frequency of microbial communities were lower in the ON zone than in the OUT zone; this result was consistent with those of the alpha diversity analysis (Table 3) and bar plot at the phylum level (Figure 2).

### Analysis of Similarity of Bacterial and Fungal Community Structures of Fairy Ring Soil

The PCA presented the community composition of both bacteria (Supplementary Figure 3A) and fungi (Supplementary Figure 3B) in the soil samples. PC1 explains 81.7% (bacteria) and 84.1% (fungi) of the overall variance, and PC2 explains 8.2% (bacteria) and 0.92% (fungi) of the overall variance. The results revealed that for both bacteria and fungi, samples of Q1and Q3 had a closer distance, those of Q1W and Q3W had a closer distance, and those of Q1 and Q3 were seriously separated from those of Q1W and Q3W, indicating that the same zones have similar microbial community structures but different zones were varied extremely (Supplementary Figure 3). The Adonis test revealed a significant difference among Q1, Q2, Q1W, and Q3W microbial community compositions (bacteria, *F* = 3.8875, R^2^ = 0.5931, ^**^p = 0.003; fungi, *F* = 4.8671, R^2^ = 0.646, ^**^p = 0.007).

### Correlation Between Soil Properties and Microbial Community Structures of Fairy Ring Soil

**FIGURE 4:**
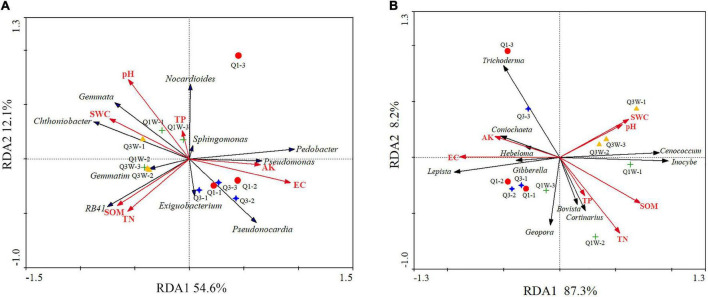
Redundancy analysis (RDA) ordination biplot showing the relationships between soil properties and bacterial and fungal communities different samples of fairy ring. **(A)** bacteria; **(B)** fungi. AK, available K; TN, total N; TP, total P; SOM, soil organic matter; WC, water content; EC, electronical conductivity.

Redundancy analysis was used to explore the association among sample soil properties; the top 10 genera (bacteria and fungi); and soil samples of Q1, Q3, Q1W, and Q3W. RDA1 and RDA2 explained 54.6% and 12.1% of total variation in the bacterial community structure, respectively (Figures 4A,B). Monte Carlo significance tests revealed that the bacterial community composition significantly correlated with EC (*F* = 9.22, *p = 0.002, variance explained = 48%) (Table 4). To some extent, the bacterial community structures of Q1 and Q3 were positively correlated with soil AK and EC; however, those of Q1W and Q3W were positively correlated with pH, SW, SOM, and TN (Figure 4A). For example, SWC and pH can affect bacterial genera *Gemmata* and *Chthoniobacter*, and SOM and TN affected *RB41*; these were the dominant genera in the OUT samples.

**TABLE 4:** Correlation analysis of soil physicochemical properties and microorganisms of fair ring by Monte Carlo permutation test.

Variable factors	Bacteria			Fungi		
		
	*p*-value	F ratio	Variance explained%	*p*-value	F ratio	Variance explained%
EC	0.002**	9.22	48	0.002**	16.33	62
pH	0.064	3.28	31	0.148	1.93	26
AK	0.278	1.33	27	0.746	0.34	27
SOM	0.684	0.62	25	0.022*	4.96	42
TP	0.318	1.21	24	0.272	1.38	26
TN	0.506	0.82	21	0.100	2.33	27
SWC	0.850	0.26	2	0.446	0.84	31

EC, electrical conductivity; pH, Soil acidity and alkalinity; AK, available potassium; SOM, soil organic matters; TP, total phosphorus; TN, total nitrogen; SWC, soil water content; *P-value < 0.05 indicates significant difference; **p-value < 0.01 indicates extremely significant difference.

For fungi, RDA1 and RDA2 explained 87.3% and 8.2% of total variation, respectively (Figure 4B). The influence of soil properties on the fungal community structure decreased in the order of EC > SOM > SWC > AK > TN > pH > TP. EC and SOM were significantly related to the fungal community structure compared with other soil properties (EC, *F* = 16.33, ^**^p = 0.002, variance explained = 62%; SOM, *F* = 4.96, *p = 0.022,% variance explained = 42) (Table 4). The fungal community structure of Q1 and Q3 had a positive relationship with EC and AK. Q1W and Q3W samples had a close positive relationship with SWC and pH but a negative relationship with EC and AK. In addition, EC and AK had a positive relationship with *Lepista*, *Trichoderma*, and *Coniochaeta*, and the fungal community structure of Q1 and Q3 samples.

### Isolation and Identification of Plant Growth-Promoting Rhizobacteria Strain and Its Effect on Plant Growth Under Salt Stress

#### Isolation of Plant Growth-Promoting Rhizobacteria From Fairy Ring Soil

We isolated 7 bacterial strains from the ON zone of fairy ring soil. The qualitative test results of the PGP function showed that BG-5 can solubilize phosphorus, produce potassium-dissolving circles on screening plates, and synthesize IAA (39.7 mg⋅mL^–1^) and siderophores (Supplementary Table 1). The strain BG-5 was selected for the next analysis.

#### Effects of Strain *Bacillus Pumilus*-5 on Plant Growth Under Different Salt Stress

**FIGURE 5:**
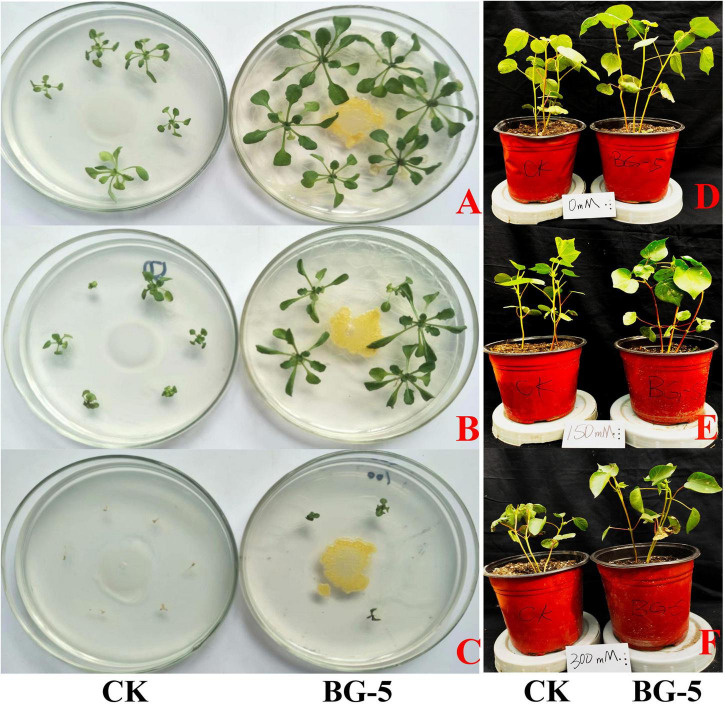
Effect of BG-5 on the growth of *Arabidopsis thaliana* and cotton under salt stress (30 days and 45 days, respctively). **(A)** *A. thaliana* treated with 0 mmol⋅L^–1^ NaCl; **(B)** *A. thaliana* treated with 50 mmol⋅L^–1^ NaCl; **(C)** *A. thaliana* treated with 50 mmol⋅L^–1^ NaCl; **(D)** cotton treated with 0 mmol⋅L^–1^ NaCl; **(E)** cotton treated with 150 mmol⋅L^–1^ NaCl; **(F)** cotton treated with 300 mmol⋅L^–1^ NaCl. Left side is CK, no-inoculated with BG-5; right side is experienment group, inoculated with BG-5.

Thirty days after the plate growth promotion test, strain BG-5-inoculated *A. thaliana* had a better growth status than no-inoculated (CK) at 0 and 50 mmol⋅L^–1^ NaCl; BG-5 had an obvious growth-promoting effect. Salt stress completely inhibited the growth of non-inoculated *A. thaliana* at 100 mmol⋅L^–1^ NaCl. However, to some extent, BG-5 inoculation increased the resistance of *A. thaliana* to salt stress (Figure 5).

Under 0 mmol⋅L^–1^ NaCl treatment, wheat inoculated with BG-5 exhibited higher dry weight (increased by 120%), fresh weight (19.5%), plant height (2.8%), root length (9.3%), and proline content (22.1%) than wheat inoculated with CK. Under 200 mmol⋅L^–1^ NaCl treatment, wheat inoculated with BG-5 exhibited higher dry weight and root length than wheat inoculated with CK (Table 5). Under 0 mmol⋅L^–1^ NaCl treatment, BG-5 improved the growth state of cotton (*Gossypium hirsutum*) compared with the control group (Figures 5E–G), and increased the leaf area, shoot length, root length, and proline and chlorophyll content by 12.7%, 8.84%, 17.50%, 54.50%, and 7.10%, respectively. Under 150 mmol⋅L^–1^ NaCl treatment, BG-5 increased the leaf area, root length, and proline and chlorophyll content of cotton; under 300 mmol⋅L^–1^ NaCl treatment, BG-5 significantly increased cotton root length and proline content to 17.4% and 27.6%, respectively. (Table 6). With increased salt stress, the POD activity was significantly higher with BG-5 than with CK; the activity increased to 66.97% and 13.02% under 150 and 300 mmol⋅L^–1^ NaCl treatments, respectively.

**TABLE 5:** The effects of BG-5 strain on wheat growth under 0 and 200 mmol⋅L^–1^ NaCl treatments.

Item	Group	Different concentration treatment of NaCl			
		
		0 mmol⋅L^–1^	N	200 mmol⋅L^–1^	N
Dry weight (g)	CK	0.15 ± 0.05b	–	0.13 ± 0.04a	
	BG-5	0.33 ± 0.43a	120%	0.16 ± 0.04a	23.1%
Fresh weight (g)	CK	0.41 ± 0.08a	–	0.44 ± 0.12a	–
	BG-5	0.49 ± 0.06a	19.5%	0.41 ± 0.02a	–6.8%
Shoot length (cm)	CK	13.33 ± 1.70a	–	11.93 ± 0.21a	–
	BG–5	13.70 ± 0.56a	2.8%	11.80 ± 1.14a	–1.1%
Root length (cm)	CK	23.60 ± 3.81b	–	21.90 ± 0.36a	–
	BG-5	25.83 ± 2.36a	9.3%	26.63 ± 0.31b	11.4%
Chlorophyll (mg/g)	CK	8.39 ± 0.42a	–	9.03 ± 0.57a	–
	BG–5	7.38 ± 0.42a	–12.0%	7.27 ± 0.40a	–19.5%
Proline (mg/g)	CK	2.31 ± 0.05a	–	12.92 ± 0.11a	–
	BG-5	2.82 ± 0.05a	22.1%	12.47 ± 0.06a	–3.5%

*N indicates the percentage increase of the treatment group compared with CK. The different letters indicate the statistical significant difference (P < 0.05) among different samples of same items and species, based on independent sample t-test.*

**TABLE 6:** The effects of BG-5 strain on cotton growth under 0, 150, and 300 mmol⋅L^–1^ NaCl treatments.

Item	Group	Different concentration treatment of NaCl					
		
		0 mmol⋅L^–1^	N	150 mmol⋅L^–1^	N	300mmol⋅L^–1^	N
Leaves area (cm^2^)	CK	36.57 ± 3.52a	–	35.74 ± 7.18a	–	25.33 ± 5.73a	–
	BG-5	41.21 ± 6.78a	12.70%	40.42 ± 6.41a	13.09%	23.44 ± 6.62a	–7.47%
Shoot length (cm)	CK	22.28 ± 1.41a	–	16.83 ± 1.64a	–	14.77 ± 1.31a	–
	BG-5	24.25 ± 2.13a	8.84%	16.75 ± 1.62a	–0.49%	14.00 ± 1.46a	–5.19%
Root length (cm)	CK	24.15 ± 1.21b		22.45 ± 0.74b	–	16.64 ± 1.20b	–
	BG–5	28.37 ± 1.10a	17.47%	25.08 ± 0.91a	11.71%	19.55 ± 0.62a	17.49%
Chlorophyll (mg⋅g^–1^)	CK	8.37 ± 1.47a	–	10.08 ± 0.79a	–	9.57 ± 1.03a	–
	BG-5	8.96 ± 0.25a	7.05%	10.62 ± 0.08a	5.36%	8.50 ± 0.72a	–11.18%
Proline content (μg⋅g^–1^)	CK	89.27 ± 9.26b	–	211.60 ± 21.95a	–	614.17 ± 68.68b	–
	BG-5	137.90 ± 15.56a	54.48%	226.70 ± 18.64a	7.13%	795.91 ± 53.10a	29.59%
Peroxidase (U⋅g^–1^⋅min^–1^)	CK	65.27 ± 5.12a	–	28.67 ± 3.11b	–	47.07 ± 8.61a	–
	BG-5	45.27 ± 4.20b	–30.64%	47.87 ± 9.09a	66.97%	53.20 ± 7.60a	13.02%

*N indicates the percentage increase of the treatment group compared with CK. The different letters indicate the statistical significant difference (P < 0.05) among different samples of same items and species, based on independent sample t-test.*

#### Phylogenetic Analysis of Selected Strains

The phylogenetic analysis of the 16S rRNA gene sequence of BG-5 revealed that the sequence was 98.82% similar to that of *Bacillus pumilus* ATCC 7061; therefore, BG-5 was identified as *B. pumilus*, and the accession number was obtained from NCBI: MK016484 (Supplementary Figure 4).

## Discussion

### *Leucocalocybe mongolica* Affected Soil Properties to Indirectly Drive Vigorous Plant Growth on Fairy Ring Soil

As the largest potential medium in nature, soil contains numerous microorganisms. Microbes can affect soil properties and provide nutrients for plant growth. In our study, we found SWC concentration were significantly declined in the ON zone due to *Leucocalocybe mongolica* growth (*p* < 0.05), that was consistent with the results of Edwards’ study (Edwards, 1988). Soil heavily infested with mycelia has a dense mesh of fungi on the surface that blocks moisture infiltration; this exactly coincides with the hypothesis regarding fairy ring formation described by Shantz (Shantz and Piemeisel, 1971). In addition, the soil pH was declined but AK and EC were significantly accumulated (*p* < 0.05) were observed in our study. That may be caused by the secreted organic acid decreased the soil pH, and organic matter was mineralized with *L. mongolica* growth, thereby increasing the soil EC. Thus, plants can absorb large amounts of directly available nutrients from the soil and grow luxuriantly (Fidanza et al., 2010). K, a critical element for plant growth and development, is involved in enzyme activation, photosynthesis, and regulation of water balance, and its accumulation strongly promotes plant growth on the fairy ring soil (Huang et al., 2020). Therefore, *L. mongolica* growth could affect the soil physicochemical properties of the ON zone, possibly driving the luxuriant growth of plants on the fairy ring soil to some extent.

### *Leucocalocybe mongolica* Affected the Soil Microbial Community Structure to Indirectly Drive Vigorous Plant Growth on the Fairy Ring Soil

Soil microbes play a vital role in maintaining ecological balance and material recycling (Hao et al., 2020). *L. mongolica* can shape soil bacterial and fungal community structures and establish a potential resource library of PGP microbes, similar to the “rhizosphere effect” of plants (Joner et al., 2001; Hinsu et al., 2021). We used high-throughput sequencing to analyze the difference of bacterial and fungal community structures and diversity between ON and OUT zones of *L. mongolica* fairy rings. According to alpha diversity analysis, the truth of the community diversity and richness of the OUT zone was higher than that of the ON zone. This similar phenomenon appeared in fairy rings formed by other fungi, such as *T. matsutake* (Kim et al., 2013) and *Lepista sordida* (Ito et al., 2017). Existence of fungi led to nutrition competition with other microbes and great diversification of ON soil properties, thereby remarkably influencing the microbial community structure and diversity (Xing et al., 2018). Many studies have reported that fairy rings can release hydrogen cyanide, affecting the number and function of dominant microbes (Bernier et al., 2016; Chen et al., 2016).

The fairy ring soil has many potential helper microorganisms (Yang et al., 2018). In our study, high throughput sequencing was used to analyzed *L. mongolica*-induced changes in the bacterial and fungal community structures. We found that Bacteroidetes seemed to play a vital role in the ON zone as evident through their higher abundance (Figure 2B). We speculated that the ON zone had a considerable number of potential PGP bacteria belonging to Bacteroidetes. Bacteroidetes are widely distributed in nature, such as sediments (Qu and Yuan, 2008), honey bee (Moran and Kwong, 2016), and forest soil (Zhang et al., 2009). Because of their ability of specifically degrade complex organic matter in the biosphere, especially polysaccharides (Kirchman, 2008). Bacteroidetes decompose plant residues in the soil and convert them into inorganic forms that can be more easily absorbed by plant, result in driving the plant flouring growth. But also, they can provide conditions for the growth of *L. mongolica* mycelia and fruiting bodies, promote the formation of fairy rings. Verrucomicrobia is widely distributed in terrestrial and aquatic ecosystems (Fierer et al., 2013; Sharp et al., 2014). In our study, we noticed that the abundance of Verrucomicrobia was lower in ON zone than OUT zone (Figure 2A). Shen et al. (2013) clarified that pH can influence Verrucomicrobia growth. We observed a lower soil pH value in ON zone than OUT zone, that may cause by rich 1 from dead vegetation, a low soil pH decreased the abundance of Verrucomicrobia. At the genus level, our research indicated that the abundance of *Pseudomonas* was higher in the ON zone than in the OUT zone (Figure 3A); similar results were noted for the *Agaricus lilaceps* fairy ring analyzed by a previous study (Caesar-Tonthat et al., 2013). Their function of adhering and binding soil particles is beneficial for soil remediation and shaping a beneficial rhizosphere environment for plant growth. In our study, we observed that *Trichoderma* was the second most dominant fungus in the soil of the *L. mongolica* fairy ring. Therefore, we can presume that *Trichoderma* has some positive influence on fairy ring formation and plant flouring growth. Some studies have indicated that *Trichoderma* metabolites have beneficial interactions with plants but have a negative effect on other fungi. They showed biocontrol activity in suppressing phytopathogenic fungi through their cell wall-degrading enzymes and secondary metabolites (Oh and Lim, 2018). In addition, our study found that *Coniochaeta* have a higher abundance in ON zone than OUT zone (Figure 3B). We educed that *Coniochaeta* has an indelible contribution to the flourishing growth of plants in the fairy ring. Many researchers have proved that *Coniochaeta* can strongly inhibit various phytopathogens by secreting ethyl acetate (Nasr et al., 2018) and improve the resistance of plants to pathogens. That just confirmed our speculated. Arbuscular mycorrhizae are symbiotic associations between plants and fungi, with their hyphae extending into the rhizosphere forming a hartig net or hyphal sheath to improve the absorption of water and nutrients such as phosphate and nitrogen (Hata et al., 2010). In addition, AMF endow host plants with tolerance to pathogens and abiotic stress (Jiang et al., 2017; Nele et al., 2020). Figure 3B presented higher fungal richness and diversity in the OUT zone than in the ON zone, because of the influence of *L. mongolica* caused dominant microbial community structures showed comparatively single in ON zone. In our study, the result showed that the abundance of ectomycorrhizal and arbuscular mycorrhizae was lower in ON zone than OUT zone, but the growth state of plants is significantly better in ON zone than OUT zone. That may be due to fungi compete with each other for nutrients and water, and space for mycelial growth (Smith and Read, 2008); this explains why OUT has a higher AMF abundance than ON in our study. Soil bacteria such as *Paenibacillus* and *Bacillus* have been reported that they can inhibit the formation of ectomycorrhizal mycorrhizae (Xing et al., 2018). Our study indicated that there may be a large number of unknown PGP microbial resources in the fairy ring soil, which still needs to be developed and excavated in the future.

#### Soil Properties and Microbial Community Structure Combine to Drive Plant Growth

Our study indicated that the abundances of the dominant genera (bacteria and fungi) were significantly correlated with the soil nutrient levels. The soil physiochemical properties varied significantly with *L. mongolica* development. These changes may influence the habitat for other native organisms. Figure 4 shows that AK and EC were positively correlated with the bacterial and fungal community structures in the ON zone, while SWC, pH, SOM, TN, and TP were positively correlated with the microbial community structure in the OUT zone. In addition, we also found that, such as *Lepista* and *Coniochaeta, as the* dominant fungal genera from the ON zone (Figure 3B), is a crucial community that participates in fairy ring formation. We also demonstrated that they were highly related to AK and EC (Figure 4B). Zhang demonstrated that available K is crucial for forming the bacterial community structure (Zhang et al., 2016a,b). Therefore, we speculated that with the growth of fairy ring-forming fungi, the K content of the ON zone of fairy ring is significantly increased, that promoted the accumulated of *Lepista* and *Coniochaeta*. These genus as PGP fungi, they promoted plant growth in ON zone and form a dense plant belt by producing secondary metabolites. *Coniochaeta ligniaria* has been confirmed as beneficial for successful colonization of some antagonistic bacteria against some phytopathogenic fungi (Kokaew et al., 2011). Additionally, *C. ligniaria* can produce 12 antifungal fatty acids to improve plant resistance against phytopathogenic fungi (Rosa et al., 2013). Trifonova demonstrated that *Coniochaeta* can stimulate growth and translocation of a consortium of PGP bacteria (Trifonova et al., 2010). This explains the phenomenon of a flourished plant band.

#### In-Depth Exploration of Plant Growth-Promoting Microbial Resources From Fairy Ring Soil

Rhizosphere is a crucial area for intensive biochemical interactions and exchanges of signal molecules between plants, soil, and microorganisms (Shaikh et al., 2018). Plant roots provide sufficient carbon and nitrogen sources to the rhizosphere, as well as establish a stable environment for microbial survival in the rhizosphere soil. PGPR with various PGP traits help to promote growth and are vital for maintaining crop growth and health under adverse environmental conditions (Khatoon et al., 2020; Grover et al., 2021). Therefore, we hypothesized that PGPR on the fairy ring soil exert a larger fraction of the driving force for vigorous plant growth.

In our study, we isolated a *B. pumilus* strain BG-5 from fairy ring soil through traditional culturable methods. Our result showed that the strain BG-5 have the PGP activities of dissolving phosphorus and potassium, producing IAA, NH_4_^+^ and siderophores. *Bacillus*, a PGPR, is widely present as plant endophytic bacteria or rhizobacteria. In many studies, *Bacillus*-containing biological products significantly promoted the growth of bell pepper roots and increased yield, while improving N fertilizer utilization (Huang et al., 2020). Meng et al. (2016) showed that *B. velezensis* BAC03 can produce IAA, ammonia, and 1-aminocyclopropane-1-carboxylate deaminase, thus promoting the growth of plants, such as beet, carrot, and cucumber. Our study results are consistent with the aforementioned results. In our study, we indicated that plants inoculated with BG-5 had higher biomass than that not inoculated. In addition, plate and pot experiment result showed that strain BG-5 could increase plant biomass, improve plant antioxidant enzyme activity and increase osmotic substances in plant cells under salt stress, so as to enhance *Arabidopsis thaliana*, wheat and cotton with salt tolerance. We speculate that *B. pumilus* strain BG-5 increases the content of available phosphorus and Fe for plants absorbed and utilized through dissolving insoluble P and enrich iron in plant rhizosphere. In addition, P is a crucial element for plant growth and development. It is pivotal for photosynthesis, cell division, signal transduction, and the synthesis of adenosine triphosphate (ATP), phospholipids, deoxyribonucleic acid (DNA), and ribonucleic acid (RNA) (Rawat et al., 2021). Therefore, we also observed that the inoculation of strains BG-5 improved the resistance of plants to salt stress. Our results also found that strain BG-5 can produce IAA, that regulated the growth and development of plants and promoted plant growth. Therefore, we also observed that the inoculated plants showed a significant increase in biomass. Proline, a plant intracellular substance, which played a role in water conservation and improved the ability of plants to resist salt stress (Kumawat et al., 2022). In our study, we also observed that the proline content of plant leaves increased significantly under salt stress after inoculating strains BG-5, the plant showed a strong salt tolerance after inoculating strain BG-5. In addition, our results showed that the activity of antioxidant enzymes (POD) in cotton leaves increased significantly under salt stress (Hassan and Beattie, 2018; Etesami and Glick, 2020). Therefore, we inferred that the strain BG-5 may eliminate oxygen free radicals in plants by induced systemic resistance (ISR) of plant, so as to reduce the toxicity of reactive oxygen and improve the tolerance of plants under salt stress (Kushwaha et al., 2019). Shahzad indicated that *B. pumilus* can improve growth performance, biomass content, antioxidase content, and tolerance against cadmium stress in maize (Shahzad et al., 2021). In addition, Kumar indicated that *B. pumilus* has various plant growth promoters, and inoculation of the selected strain leads to a positive adaptation and improve the growth performance of rice, such as plant height, root length, chlorophyll content, and antioxidant enzyme activities (Kumar et al., 2020). In conclusion, there are a large number of PGP bacteria resources in the fairy ring soil. They can improve the absorption of nutrient elements and enhance the resistance of plants to biological and biological stress through bacteria PGP function activities, so as to promote plant growth. In our study, *Bacillus* abundance in the ON zone was higher than that in the OUT zone and *Bacillus* was not the dominant genus in all samples, just accounted for 0.36% microorganisms (Figure 3A), indicating much low abundance species in fairy ring soil played an important role for flourishing growth of plant and need to be exploited and utilized.

## Conclusion

Our study indicated that fairy ring-forming fungi increased soil EC and AK content, and decreased the microbial (bacteria and fungi) diversity beneath fairy ring, shaped a beneficial microorganisms structure, these changes of micro-environment of plant rhizosphere driving the flourishing plant growth. In addition, we isolated a *B. pumilus* strain BG-5 can improve plant growth performance, enhanced salt resistance by multiple PGP properties. Our research indicated that there are a large number of beneficial microbial resources present in the fairy ring soil need to be exploited. Our study provides a good PGP candidate for use in agricultural production.

## Data Availability Statement

The datasets presented in this study can be found in online repositories. The names of the repository/repositories and accession number(s) can be found below: National Center for Biotechnology Information (NCBI) BioProject database under accession number PRJNA762433.

## Author Contributions

QW designed and performed the experiments, analyzed the data, and drafted the manuscript. CW and YW helped with data analysis, and revised and refined the manuscript. YL collected the samples and analyzed part of the data. YW participated in data analysis and drafting of the manuscript. YS designed and performed the experiments and analyzed the data. All authors read and approved the final version of the manuscript.

## Conflict of Interest

The authors declare that the research was conducted in the absence of any commercial or financial relationships that could be construed as a potential conflict of interest.

## Publisher’s Note

All claims expressed in this article are solely those of the authors and do not necessarily represent those of their affiliated organizations, or those of the publisher, the editors and the reviewers. Any product that may be evaluated in this article, or claim that may be made by its manufacturer, is not guaranteed or endorsed by the publisher.

## Footnotes

1
https://www.cpc.ncep.noaa.gov/

2
https://geodata.pku.edu.cn/index.php?c=content&a=show&id=416#

3
http://drive5.com/uchime/uchime_download.html

4
http://www.drive5.com/usearch/manual/uchime_algo.html

5
https://www.arb-silva.de/

6
https://unite.ut.ee/

## Supplementary Material

Supplementary Figure 1 Rarefaction curves based on the sequences of the V3-V4 region of the 16S rRNA gene and ITS region amplified of the fungi eukaryotic ribosomal RNA gene from fairy rings samples. The abscissa indicates the number of extracted tags; the ordinate indicates the expected number of OTUs that can be obtained when a certain number of tags were extracted. **(A)** bacteria; **(B)** fungi.

Supplementary Figure 2 Venn diagram showing the common and unique bacterial and fungal OTUs among different samples associated with OUT and ON zone from fairy rings. **(A)** bacteria; **(B)** fungi.

Supplementary Figure 3 Principal component analysis (PCA) was used to compared of microbial community structure between different samples of fairy rings in OTUs level (at 97% similarity). **(A)** bacteria; **(B)** fungi.

Supplementary Figure 4 Phylogenetic analysis of the 16s rRNA gene sequence of BG-5. The phylogenetic tree was constructed using the neighbor-joining method. Bootstrap analysis with 1000 replications was performed to assess the topology of the phylogenetic trees. The evolutionary distances were calculated using Kimura’s two-parameter model.

Supplementary Table 1 Qualitative testing of plant growth promoting function of isolated strains from fairy ring soil. Positive result of plant-growth-promoting activity are indicated by “ + ”, negative result of plant-growth-promoting activity are indicated by “−”.
