# Supplementary figures and images for: Soil Microbes Drive the Flourishing Growth of Plants From Leucocalocybe mongolica Fairy Ring
Source: Front Microbiol. 2022 May 20;13:893370. doi: 10.3389/fmicb.2022.893370 (PMC9164162; doi:10.3389/fmicb.2022.893370)

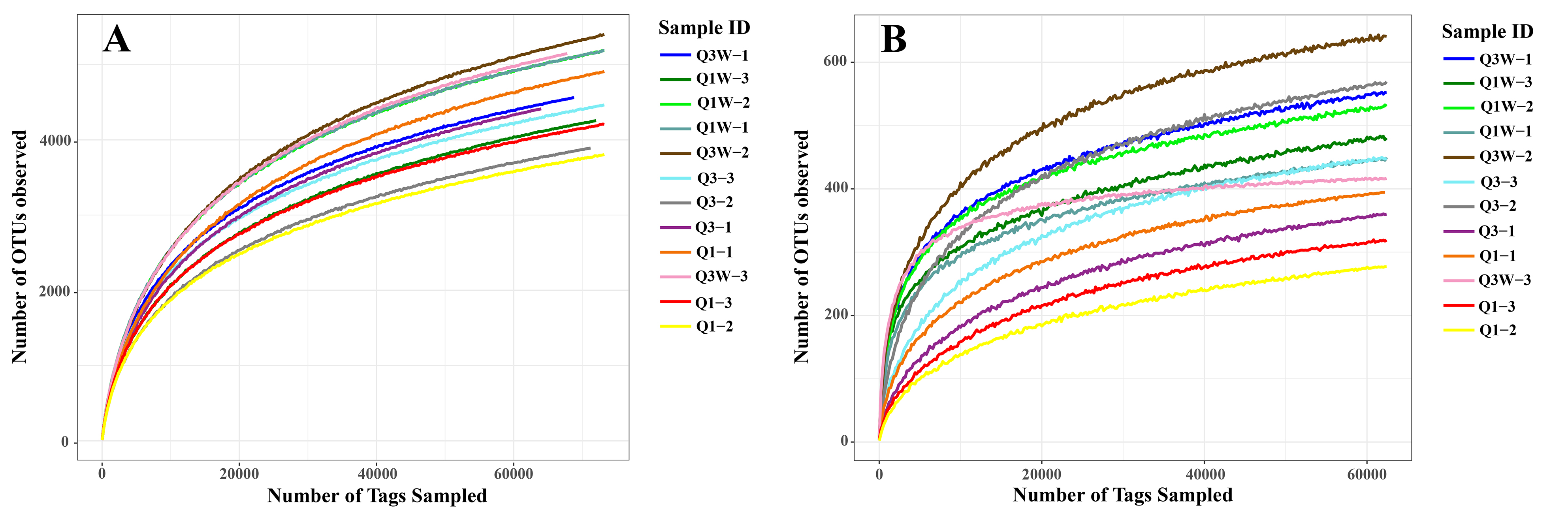

Supplement: Supplementary Figure 1 — Rarefaction curves based on the sequences of the V3-V4 region of the 16S rRNA gene and ITS region amplified of the fungi eukaryotic ribosomal RNA gene from fairy rings samples. The abscissa indicates the number of extracted tags; the ordinate indicates the expected number of OTUs that can be obtained when a certain number of tags were extracted. (A) bacteria; (B) fungi. [file Image_1.JPEG]

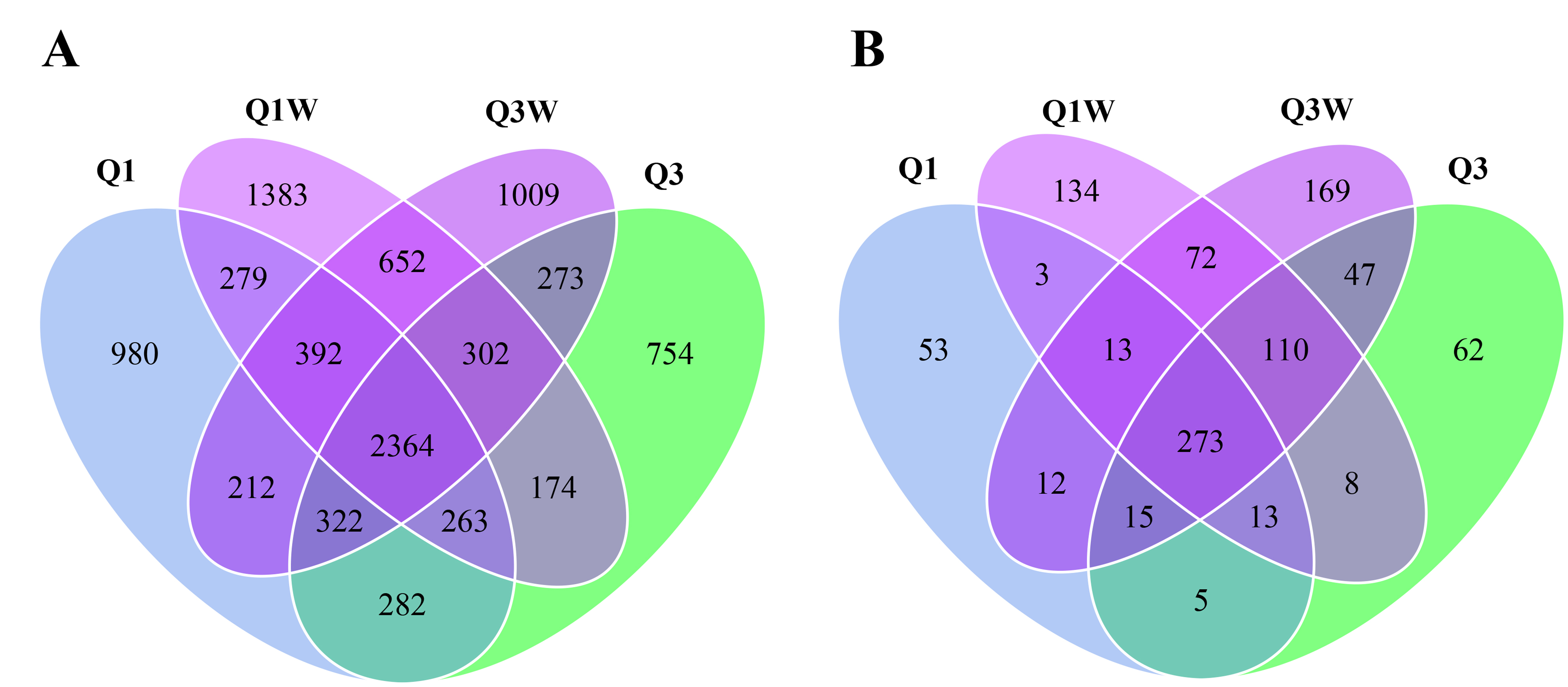

Supplement: Supplementary Figure 2 — Venn diagram showing the common and unique bacterial and fungal OTUs among different samples associated with OUT and ON zone from fairy rings. (A) bacteria; (B) fungi. [file Image_2.JPEG]

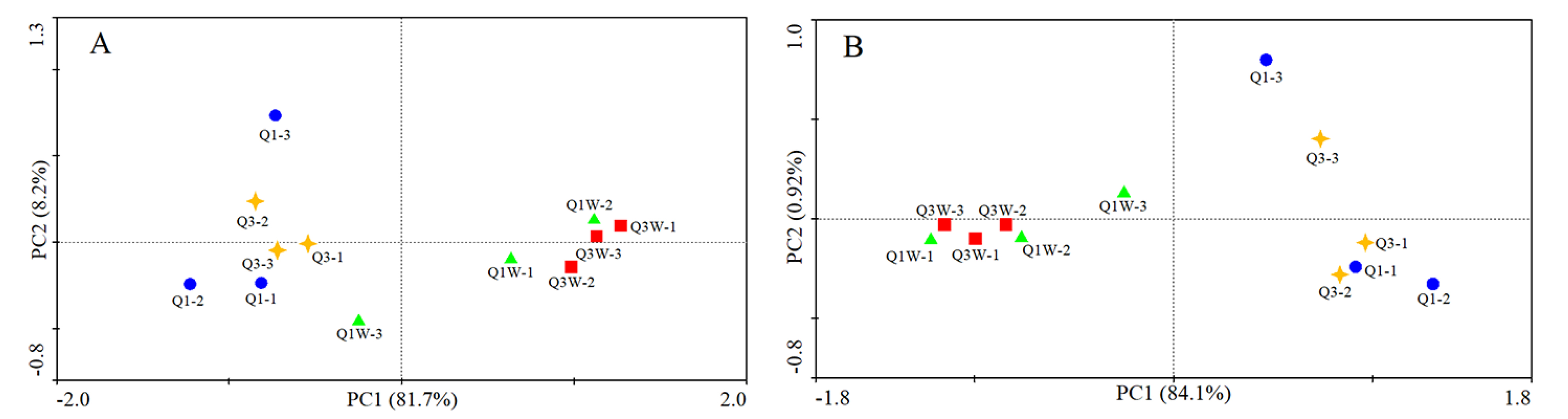

Supplement: Supplementary Figure 3 — Principal component analysis (PCA) was used to compared of microbial community structure between different samples of fairy rings in OTUs level (at 97% similarity). (A) bacteria; (B) fungi. [file Image_3.JPEG]

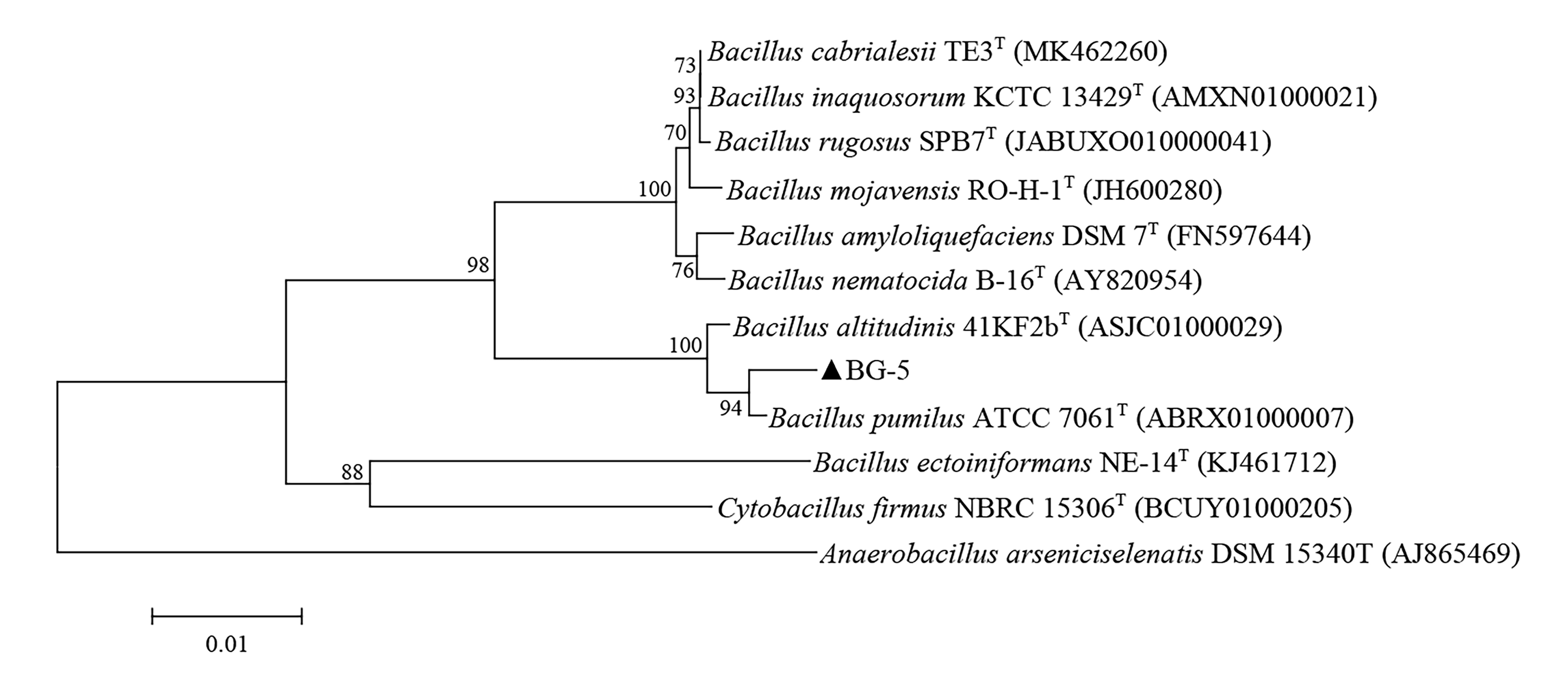

Supplement: Supplementary Figure 4 — Phylogenetic analysis of the 16s rRNA gene sequence of BG-5. The phylogenetic tree was constructed using the neighbor-joining method. Bootstrap analysis with 1000 replications was performed to assess the topology of the phylogenetic trees. The evolutionary distances were calculated using Kimura’s two-parameter model. [file Image_4.JPEG]
