# Supplementary material for: Soil Microbes Drive the Flourishing Growth of Plants From Leucocalocybe mongolica Fairy Ring
Source: Front Microbiol. 2022 May 20;13:893370. doi: 10.3389/fmicb.2022.893370 (PMC9164162; doi:10.3389/fmicb.2022.893370)
Supplement: Supplementary Table 1 — Qualitative testing of plant growth promoting function of isolated strains from fairy ring soil. Positive result of plant-growth-promoting activity are indicated by “ + ”, negative result of plant-growth-promoting activity are indicated by “−”. [file Table_1.DOCX]

| Table S1 Qualitative testing of plant growth promoting function of isolated strains from fairy ring soil. Positive result of plant-growth-promoting activity are indicated by “+”, negative result of plant- growth-promoting activity are indicated by “-”. | | | | | | |
| --- | --- | --- | --- | --- | --- | --- |
|  | Nitrogen fixation capacity | Phosphorus solubilization capacity | Potassium dissolving capacity | IAA production capacity  (mg·mL^-1^) | NH_4_^+^  production capacity | Siderophores  production  capacity |
| BG-1 | + | + | + | - | - | - |
| BG-2 | - | - | - | - | + | - |
| BG-3 | - | + | - | - | - | + |
| BG-4 | + | - | - | - | - | - |
| BG-5 | - | + | + | 39.7 | + | + |
| BG-7 | + | - | - | - | + | - |
| BG-10 | - | - | + | - | + | - |
